# **Intratumor lactate levels reflect HER2 addiction status in HER2‐positive breast cancer**

**DOI:** 10.1002/jcp.27049

**Published:** 2018-08-21

**Authors:** Lorenzo Castagnoli, Egidio Iorio, Matteo Dugo, Ada Koschorke, Simona Faraci, Rossella Canese, Patrizia Casalini, Patrizia Nanni, Claudio Vernieri, Massimo Di Nicola, Daniele Morelli, Elda Tagliabue, Serenella M. Pupa

**Affiliations:** ^1^ Molecular Targeting Unit, Department of Research Fondazione IRCCS Istituto Nazionale dei Tumori Milan Italy; ^2^ Core Facilities, NMR Unit Istituto Superiore di Sanità Roma Italy; ^3^ Functional Genomics and Bioinformatics Core Facility, Department of Applied Research and Technology Development, Fondazione IRCCS Istituto Nazionale deiTumori Milan Italy; ^4^ Laboratory of Immunology and Biology of Metastasis, Department of Experimental, Diagnostic and Specialty Medicine University of Bologna Bologna Italy; ^5^ IFOM, FIRC Institute of Molecular Oncology Milan Italy; ^6^ Department of Medical Oncology and Hematology, Fondazione IRCCS Istituto Nazionale dei Tumori Milan Italy; ^7^ Unit of Immunotherapy and Anticancer Innovative Therapeutics, Department of Medical Oncology and Hematology Fondazione IRCCS Istituto Nazionale dei Tumori Milan Italy; ^8^ Laboratory Medicine Unit, Department of Pathology and Laboratory Medicine Fondazione IRCCS Istituto Nazionale dei Tumori Milan Italy

**Keywords:** breast cancer, glycolysis, HER2, lactate, oncogene addiction

## Abstract

Despite different molecular tumor profiles indicate that human epidermal growth factor receptor 2 (HER2) messenger RNA (mRNA) levels mirror HER2 addiction and trastuzumab benefit in HER2‐positive breast cancer (BC), the identification of noninvasive clinical predictors of trastuzumab sensitivity remains an unmet clinical need. In the current study, we investigated whether intratumor lactate levels reflect HER2 addiction and, in turn, trastuzumab susceptibility. Accordingly, the gene expression profiles of transgenic murine BC cell lines expressing the human d16HER2 variant (HER2‐addicted) or human full‐length HER2 (WTHER2; HER2‐nonaddicted) revealed a significant enrichment of glycolysis‐related gene pathways in HER2‐addicted cells. We studied the metabolic content of 22 human HER2‐positive BC by quantitative nuclear magnetic resonance spectroscopy and found that those cases with higher lactate levels were characterized by higher HER2 transcript levels. Moreover, gene expression analyses of HER2‐positive BC samples from a TCGA data set revealed a significant enrichment in glycolysis‐related pathways in high/HER2‐addicted tumors. These data were confirmed by metabolic analyses of human HER2‐positive BC cell lines with high or low HER2 transcript levels, which revealed significantly more active glycolytic metabolism in high HER2 transcript than in low HER2 transcript cells. Overall, our results provide evidence for noninvasive intratumor lactate detection as a potential metabolic biomarker of HER2 addiction and trastuzumab response suggesting the possibility to use in vivo imaging to assess lactate levels and, in turn, select HER2‐positive BC patients who are more likely to benefit from anti‐HER2 treatments.

## 1 INTRODUCTION

Human epidermal growth factor receptor 2 (HER2)‐positive breast cancer (BC) is defined by high expression levels of the HER2 tyrosine kinase receptor as determined by immunohistochemistry (IHC) and/or amplification of the HER2 gene as evidenced by fluorescence in situ hybridization (FISH; Loibl & Gianni, 2017; Ménard et al., 2000). Globally, HER2‐positive BC accounts for approximately 20% of all BCs and is characterized by a highly aggressive disease course, more frequent relapses in the absence of effective adjuvant treatments, and poor survival in the metastatic setting (Loibl & Gianni, 2017; Ménard et al., 2000). The introduction of the humanized anti‐HER2 monoclonal antibody (mAb) trastuzumab in combination with chemotherapy has improved both the relapse‐free survival (RFS) and overall survival (OS) of primary and metastatic HER2‐positive BC patients (Joensuu et al., 2006; Piccart‐Gebhart et al., 2005; Pupa et al., 2005; Romond et al., 2005; Slamon et al., 2001). In recent years, different rationally designed HER2‐targeting agents have been developed and successfully tested in clinical trials. Among them, the combination of trastuzumab with lapatinib (a tyrosine kinase inhibitor) or pertuzumab (anti‐HER2 mAb) improved the pathological complete response rate in the neoadjuvant setting compared with trastuzumab alone (Baselga et al., 2012; Gianni et al., 2012), while the Aphinity trial demonstrated a reduced risk of relapse in patients who received the trastuzumab–pertuzumab combination in the adjuvant setting (Von Minckwitz et al., 2017).

Unfortunately, the response of HER2‐positive tumors to anti‐HER2 treatments is highly heterogeneous, with some patients deriving long‐term benefit and other patients bearing primarily resistant tumors (Carey, 2012). High HER2 expression, as determined by IHC score or FISH analysis, cannot be considered per se a good indicator of HER2 addiction or the response to trastuzumab or other anti‐HER2 treatments because of (a) poor performance of IHC and FISH methods currently used to define HER2‐positive BC, which do not take into account HER2 protein levels or provide suboptimal quantification of HER2 levels, and (b) molecular heterogeneity of HER2‐positive neoplasms, with some tumors relying on other growth–proliferation pathways either independent or downstream of HER2 signaling. Distinguishing between HER2‐addicted and HER2‐nonaddicted BC, that is those responsive or not responsive to trastuzumab, could help clinicians to select HER2‐positive BC patients who are more likely to benefit from anti‐HER2 therapies in the adjuvant and metastatic settings.

Recent studies have suggested that high HER2 messenger RNA (mRNA) levels are directly associated with the trastuzumab response, and thus, high HER2 mRNA levels have been proposed as a marker of HER2 addiction (Di Modica et al., 2017; Prat et al., 2014; Triulzi et al., 2015). Several preclinical studies have revealed the capability of HER2‐driven signaling to promote glucose uptake, oxygen consumption, overexpression of lactate dehydrogenase‐A (LDH‐A), and lactate production in tumor cells (Walsh et al., 2013; Zhao et al., 2009). In particular, intratumor lactate accumulation has been associated with tumor metastatic potential and patient OS and has been shown to contribute to immune escape, cell migration, and radioresistance in several studies (Hirschhaeuser et al., 2011). In addition, HER2‐induced upregulation of hypoxia‐induced factor 1α (HIF1α; Laughner et al., 2001) could mediate the burst in glycolytic metabolism that is a feature typical of these tumors (Laughner et al., 2001; Robey et al., 2005) and induces the overexpression of specific glycolysis‐related enzymes involved in the lactate production such as LDH‐A (Marin‐Hernandez et al., 2009).

This study was designed to identify tumor‐related, noninvasive biomarkers predictive of the response of HER2‐positive BC to anti‐HER2 treatment. To this aim, we investigated human HER2‐positive cancer cell lines, transgenic models, and human tumor specimens at the molecular and metabolic levels using gene expression profiling and high‐resolution magnetic resonance spectroscopy (MRS; Kang et al., 2012). Overall, our findings indicate that HER2‐addicted BCs, that is tumors that respond to anti‐HER2 treatment, are characterized by high HER2 mRNA levels and the significant upregulation of glycolytic metabolism. Our results pave the way for exploiting in vivo magnetic resonance imaging and MRS as a reliable and noninvasive clinical tool to detect intratumor lactate levels and thus selecting HER2‐positive BC patients who are more likely to benefit from anti‐HER2 treatments in both the adjuvant and metastatic treatment settings.

## 2 MATERIALS AND METHODS

### 2.1 Tumor cell lines

The human HER2‐positive BC cell lines BT474, MDAMB361, and MDAMB453 were grown in monolayer cultures in the Dulbecco’s modified Eagle’s medium (EuroClone, Pero, MI, Italy) with 10% fetal bovine serum (FBS; Sigma‐Aldrich, St. Louis, MO), whereas SKBR3, ZR75.30, and HCC1954 cells were grown in monolayer cultures in RPMI1640 medium with 10% FBS. Human tumor cell lines were obtained between 2,000 and 2,010 and authenticated by short tandem repeat DNA fingerprinting using the AmpFISTR identifier polymerase chain reaction (PCR) Amplification Kit (Thermo Fisher Scientific, Waltham, MA; last verification, November 2015). The transgenic murine primary mammary tumor cell lines MI6, MI7, WTHER2_1, and WTHER2_2, which express the human d16HER2 splice variant or full‐length wild‐type HER2 (WTHER2), have been described previously (Castagnoli et al., 2014; Castagnoli et al., 2017; De Giovanni et al., 2014); these cell lines were maintained in complete MammoCult medium (StemCell Technologies, Vancouver, BC, Canada) supplemented with 1% FBS (Sigma‐Aldrich) and penicillin‐streptomycin (Sigma‐Aldrich). All tumor cell lines were cultured at 37°C in a humidified 5% CO_2_ atmosphere and were routinely tested for mycoplasma contamination.

### 2.2 Patient cohort

The 39 HER2‐positive BC patients included in this study were part of the observational retrospective multicenter Italian study Group Herceptin in Adjuvant Therapy (GHEA; Campiglio et al., 2013; Supporting Information Table 1). Patients were treated at Fondazione IRCCS Istituto Nazionale dei Tumori with adjuvant trastuzumab between 2005 and 2009. First relapse events were registered, and RFS was calculated as the time between the initiation of trastuzumab treatment and the first event. The Independent Ethics Committee of Fondazione IRCCS Istituto Nazionale Tumori approved the molecular characterization of material from patients included in this observational study.

### 2.3 Animal models

Female Friend Virus B (FVB) mice (6–8 weeks old, body weight 20–25 g) were purchased from Charles River (Calco, Italy) and maintained in the Animal Facility of Fondazione IRCCS Istituto Nazionale dei Tumori. Animal care and experimental procedures were approved by the Ethics Committee for Animal Experimentation of the Institute according to Italian law. MI6 or WTHER2 tumor cells (1 × 10^6^) were injected into the mammary fat pad (m.f.p.) of all FVB mice (*n* = 7/group), except those in the dimethyl sulfoxide (DMSO) group (*n* = 6). When tumors reached approximately 50 mm^3^, mice were randomized into two groups to receive lapatinib at a daily dose of 200 mg/kg or diluent (DMSO) per os. Tumors were measured twice weekly, and tumor volume was calculated as 0.5 × *d*_1_^2^ × *d*_2_, where *d*_1_ and *d*_2_ are the smaller and larger tumor diameters, respectively. Mice were killed when the tumor volume reached ~2,000 mm^3^.

### 2.4 Quantitative real‐time PCR (qPCR)

Human HER2‐positive BC cell lines (*n* = 6) and frozen primary tumor specimens (*n* = 39) were analyzed by qPCR to determine HER2 and hypoxia‐inducible factor 1α (HIF1α) transcript levels as previously described (Mitra et al., 2009). Briefly, total RNA from tumor specimens was extracted using the ReliaPrep miRNA Cell and Tissue Miniprep System Kit (Promega, Fitchburg, WI) following the manufacturer’s instructions. cDNA was reverse‐transcribed from 1 µg of total RNA in a 20‐µl volume with the High‐Capacity RNA‐to‐cDNA Kit (Thermo Fisher Scientific), and 25 ng of cDNA was examined by qPCR using Applied Biosystems SYBR Green dye‐based PCR assays on the ABI Prism 7900HT sequence detection system (Applied Biosystems, Foster City, CA). HER2 and HIF1α transcripts were amplified using 200 nM primers (Mitra et al., 2009; HIF1 forward: CGTTCCTTCGATCAGTTGTC; HIF1α reverse: TCAGTGGTGGCAGTGGTAGT).

Target gene data were normalized to glyceraldehyde 3‐phosphate dehydrogenase (GAPDH) data (GAPDH forward: GCTCACTGGCATGGCCTTC; GAPDH reverse: CCTTCTTGATGTCATCATATTTGGC). The relative abundance of HER2 mRNA was calculated by the comparative *C*_t_ method (Triulzi et al., 2013).

### 2.5 Western blot analysis

Human HER2‐positive BC cell lines were solubilized for 40 min at 0°C with lysis buffer, as described (Ghedini et al., 2010). Briefly, after solubilization, the samples were mixed with gel sample buffer under reducing conditions, heated for 5 min at 95°C, and resolved by electrophoresis on precast 4–12% Bis‐Tris gels (Thermo Fisher Scientific). The separated proteins were electrophoretically transferred onto nitrocellulose membranes, which were stained with Ponceau S to check protein loading, washed extensively with Tris‐buffered saline (TBS)+ 0.5% Tween‐20 and incubated for 1 hr at room temperature in blocking solution (5% low‐fat milk in TBS + 0.1% Tween‐20) before the addition of primary antibodies in 3% low‐fat milk in TBS + 0.1% Tween‐20 for 1 hr at room temperature or overnight at 4°C with gentle shaking. The following primary mouse mAbs were used: Ab3 c‐erbB‐2/HER2/neu (1:100; Calbiochem, Darmstadt, Germany) directed to the human HER2 intracellular domain and anti‐β‐actin‐peroxidase (AC‐15 clone; 1:50,000; Sigma). The membranes were then washed extensively with TBS + 0.5% Tween‐20 and incubated with horseradish peroxidase‐conjugated goat anti‐mouse IgG (1:5,000; Amersham GE Healthcare, Little Chalfont, UK) for 1 hr at room temperature. The signals were detected using enhanced chemiluminescence (ECL; Amersham GE Healthcare).

### 2.6 Assessment of extracellular lactate levels

Extracellular lactate levels in conditioned medium from cultured cancer cells were determined by a blood gas analyzer (GEM Premier 4000; Werfen, Bedford, MA). Briefly, HER2‐positive BC cell lines were cultured in a monolayer until they reached approximately 80% confluence; 12 hr before the test, the culture medium was replaced with fresh medium. To avoid protein contamination that could alter the lactate quantification, culture media were deproteinized using Amicon Ultra centrifugal filter units (molecular weight cut‐off 10 kDa; Merck‐Millipore, Billerica, MA) following the manufacturer’s instructions. Lactate levels were normalized to the number of tumor cells.

### 2.7 Quantification of nicotinamide adenine dinucleotide (NADH) and flavin adenine dinucleotide (FAD) levels and the optical redox ratio (NADH/FAD)

Samples were imaged using a confocal laser scanning microscope (Leica TCS SP8 X; Leica Microsystems GmbH, Mannheim, Germany). The fluorochromes were excited by a 405 nm continuous wave diode laser and a pulsed supercontinuum white light laser (470–670 nm; 1‐nm tuning step size). In particular, FAD was excited by the 488 nm laser line and detected from 501 to 600 nm, while NADH was excited by a continuous wave 405 nm diode laser and detected from 410 to 495 nm. Images of 512 × 512 or 1024 × 1024 pixels were acquired during scanning using an HC PL APO 63 × /1.40 CS2 oil immersion objective and a pinhole set to 1 Airy unit. Differential interference contrast images were acquired with the 488 nm laser line. NADH levels and the optical redox ratio were quantified and analyzed using ImageJ 1.51p  (National Institutes of Health, Bethesda, MD).

### 2.8 Bioinformatic analyses

Raw gene expression data for murine mammary cell lines and human tumor specimens (GHEA cohort) were downloaded from the Gene Expression Omnibus (GEO) repository with accession numbers GSE67300 and GSE55348, respectively. Data from both datasets were log 2‐transformed and normalized using the robust spline normalization method implemented in the Bioconductor package lumi (Du et al., 2008). Only probes with a detection *p* < 0.01 in at least one sample were retained, and for each gene, the probe with the highest variance was selected. Samples in GSE55348 were assigned to PAM50 subtypes (Prat et al., 2014; basal, HER2‐enriched, luminal A, luminal B, and normal‐like), using the molecular.subtyping function of the genefu package.

The association of ERBB2 gene expression (probe ILMN_1728761) and PAM50 subtypes was assessed by Welch’s analysis of variance. The association between ERBB2 gene expression and tumor relapse was assessed using unpaired two‐tailed Student’s *t* test. The Cancer Genomic Atlas (TCGA), clinical data, and RSEM gene‐level RNAseqv2 data were downloaded from the Firehose Broad data portal (https://gdac.broadinstitute.org/, data version 2016_01_28). RSEM values were transformed into log 2 counts per million using the voom function of the limma package (Ritchie et al., 2015). Gene set enrichment analysis (GSEA) (Subramanian et al., 2005) was performed in a pre‐ranked mode using gene sets from the MSigDB C2 canonical pathway collection. For GSE67300, genes were ranked according to the t‐statistic calculated between the d16HER2 and WTHER2 cell lines using the limma package [24]. For TCGA, genes were ranked according to the Pearson’s correlation coefficient with ERBB2 gene expression levels.

### 2.9 High‐resolution MRS of the cell and tissue extracts

Aqueous extracts from 20 to 30 × 10^6^ cells grown to 60–70% confluence and tissues were prepared in EtOH:H_2_O (70:30, vol/vol) as previously described (Iorio et al., 2010). Samples were ultrasonicated at 20 kHz with an exponential probe (8 mm peak‐to‐peak) in an MSE ultrasonic disintegrator Mk2 (Crawley, Sussex, UK) and centrifuged at 14,000*g* for 30 min. Supernatants were lyophilized twice in an RVT 4104 Savant lyophilizer  (Waters Corporation, Mildford, ME). For MRS analyses, the residue was resuspended in 0.7 ml of D_2_O (Sigma‐Aldrich, Milan, Italy) containing 0.1 mM 3(trimethylsilyl)‐propionic‐2,2,3,3‐d4 acid sodium salt as an internal standard (Merck & Co, Montreal, QC, Canada).

High‐resolution MRS analyses (25°C) were performed at 9.4 T (Bruker Avance spectrometer, Karlsruhe, Germany). Spectra of the cell and tissue extracts were obtained using radio‐frequency pulses for excitation, water signal presaturation, data processing, and data analysis as described (Pisanu et al., 2014). Relative metabolite quantification was presented in nanomole normalized to the number of extracted cells or as the percentage of an individual metabolite among all metabolites.

### 2.10 Statistical analysis

Associations between categorical variables were tested using the two‐sided Fisher’s exact test. A *p* < 0.05 indicated statistical significance. Differences in continuous variables between groups were tested using a two‐tailed unpaired *t* test. Differences were considered significant at *p* < 0.05. Linear regression analysis and Pearson’s correlation coefficient *r* were used to estimate the correlations between (a) HER2 expression and lactate levels in HER2‐positive tumor specimens, (b) HER2 and HIF1α transcript levels in tumor specimens, (c) HER2 expression and NADH levels in six different HER2‐positive BC cell lines, (d) HER2 expression and the optical redox ratio in six different HER2‐positive BC cell lines, (e) HER2 expression and intracellular and extracellular lactate levels in six different HER2‐positive BC cell lines, and (f) HER2 expression and intracellular succinate levels. All analyses were performed using GraphPad Prism (version 5.02).

### 2.11 Ethics approval and consent to participate

Samples from 53 HER2‐positive patients who were diagnosed between 2005 and 2009 at our institute (Fondazione IRCCS Istituto Nazionale dei Tumori) were derived from our multicenter Italian observational study GHEA (Campiglio et al., 2013). All data were analyzed anonymously, and all procedures complied with the Declaration of Helsinki.

Samples were donated by patients to the Institutional BioBanks for research purposes, and all patients provided written consent for the use of their biological materials for future investigations. Aliquots were allocated to this study after approval by the Institutional Review Board and a specific request to the independent ethical committee of the Institute “Comitato Etico della Fondazione IRCCS Istituto Nazionale dei Tumori, Milano,” registered at AIFA (Agenzia Italiana del Farmaco‐Italian Drug Agency).

## 3 RESULTS

### 3.1 HER2 addiction is associated with a glycolytic gene expression signature in HER2‐positive transgenic preclinical models

**Figure 1:**
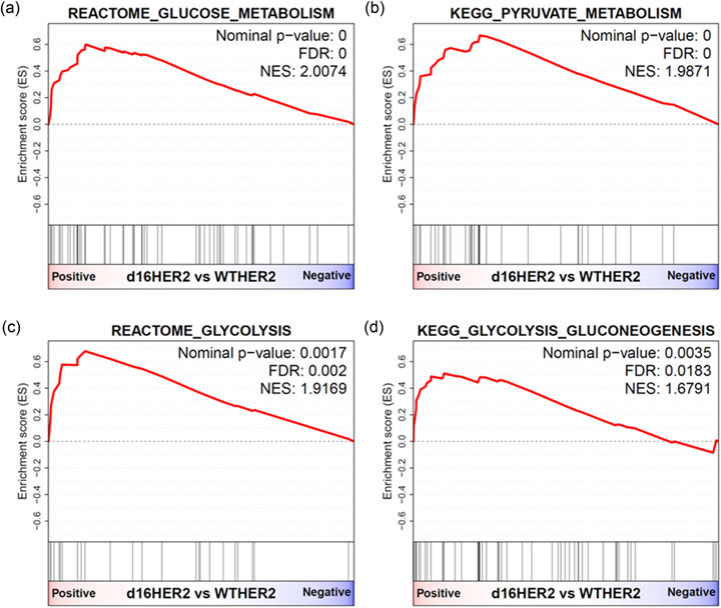
GSEA plots of significantly enriched gene sets in the comparison between active primary d16HER2‐ and WTHER2‐positive mammary tumor cells. (a–d) Plots of glucose, pyruvate metabolism, glycolysis, and glycolysis_gluoconeogenesis gene sets in transgenic MI6 and WTHER2‐positive (WTHER2_1) murine mammary tumor cells. GSEA: gene set enrichment analysis

To identify biological characteristics specific of HER2‐positive BC cells that are addicted to HER2 signaling (Shiu et al., 2014) and responsive to anti‐HER2 therapies, we analyzed the global gene expression profiles of HER2‐positive grafts obtained by injecting transgenic murine mammary cell lines expressing the human d16HER2 variant (MI6) or full‐length–wild‐type *HER2* (WTHER2_1) into the m.f.p. of parental FVB mice. Based on their response to lapatinib, and our already reported data, MI6 cells are considered a model of HER2 addiction, while WTHER2_1 cells are not addicted to HER2 (Castagnoli et al., 2014, 2017; Supporting Information Figure 1). Functional annotation through GSEA analysis revealed a significant upregulation of four different cellular pathways related to glucose metabolism in d16HER2‐positive cells compared with the corresponding WTHER2‐positive cells. In particular, genes involved in glucose and pyruvate metabolism, glycolysis and gluconeogenesis were upregulated in HER2‐addicted cells compared with nonaddicted cells (Figure 1).

### 3.2 Intratumor lactate level predicts the benefit of adjuvant trastuzumab‐containing treatments in patients with HER2‐positive BC

We then examined whether the enhanced glycolysis in HER2‐addicted preclinical BC models is mirrored in human tumor specimens (Prat et al., 2014). We first evaluated whether HER2 transcript levels in the GHEA cohort were associated with the tumor relapse in patients who received trastuzumab‐containing adjuvant therapy, as was recently suggested (Di Modica et al., 2017).

**Figure 2:**
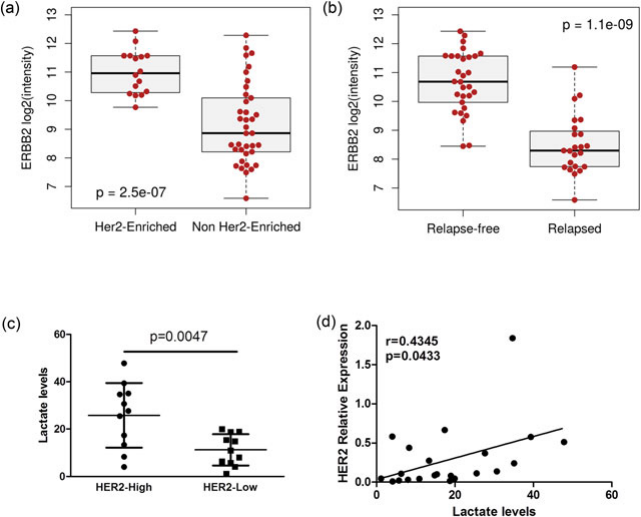
Association between HER2 transcript expression and intratumor lactate levels in human HER2‐overexpressing BC. (a) Box plots showing HER2 mRNA expression levels [ERBB2 log 2(intensity)] measured by microarray analysis in 53 HER2‐positive BC patients treated with adjuvant trastuzumab (GHEA cohort, GSE55348) distributed according to PAM50 BC classification in the HER2‐enriched versus the other merged BC subgroups (basal, luminal A, luminal B, and normal‐like). Significance was calculated by a two‐tailed unpaired *t* test. (b) Box plots showing HER2 mRNA expression levels [ERBB2 log 2(intensity)] measured by microarray analysis in 53 HER2‐positive BC patients treated with adjuvant trastuzumab (GHEA cohort, GSE55348) in Relapsed and Relapse‐free patients. Significance was calculated by a two‐tailed unpaired *t* test. (c) Association between HER2 transcript levels (high vs. low) and intratumor lactate levels by high‐resolution multinuclear MRS. (d) Pearson’s correlation between relative HER2 transcript levels and intratumor lactate levels by high‐resolution multinuclear MRS. The data are presented as the mean ± *SD*. Significance was calculated by a two‐tailed unpaired *t* test. BC: breast cancer; GHEA: Group Herceptin in Adjuvant Therapy; HER2: human epidermal growth factor receptor 2; mRNA: messenger RNA; MRS: magnetic resonance spectroscopy

In silico analysis of the gene expression profiles of 53 primary HER2‐positive specimens from the GHEA cohort (Campiglio et al., 2013) enabled to stratify HER2‐positive BC patients according to the classification of prediction analysis of microarray 50 (PAM50; basal, HER2‐enriched, luminal A, luminal B, and normal‐like molecular subtypes). Notably, the HER2‐enriched BC subtype that includes HER2‐positive BC cases more susceptible to trastuzumab treatment (Prat et al., 2014; *n* = 16) was characterized by significantly higher HER2 mRNA levels in comparison with the other BC subtypes considered all together (*n* = 37; Figure 2; *p* = 2.5 × 10^−7^) or separately (Supporting Information Figure 2; *p* = 4.6 × 10^−5^), In addition, GHEA relapsed cases had significantly lower HER2 mRNA levels than those relapse‐free (*p* = 1.1 × 10^−9^; Figure 2b).

These data suggest that HER2 mRNA levels can be used as a biomarker of clinical benefit from trastuzumab, thus potentially discriminating between HER2‐addicted and HER2‐nonaddicted cases of HER2‐positive BC. To investigate the association between HER2 addiction and glycolytic pathway activation, high‐resolution ex vivo MRS was used to assess the global metabolic profile of 22 of 53 GHEA cases. This analysis revealed a significant increase in lactate concentration in tumor specimens with higher HER2 mRNA levels (*p* = 0.0047; Figure 2c), a significant positive correlation between HER2 transcript level and intratumor lactate concentration (*p* = 0.0433 and Pearson’s *r* = 0.4345; Figure 2d). Taken together, these results suggest that HER2 mRNA and lactate levels are reliable biomarkers of BC addiction to HER2 signaling.

**Figure 3:**
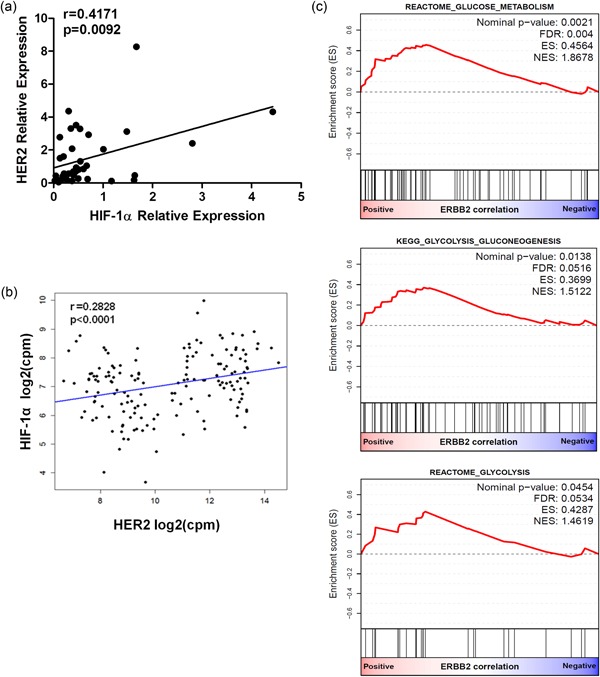
Correlation between HER2 transcript expression and glycolysis‐related genes in human HER2‐overexpressing BC. (a) qPCR analysis in cases from the GHEA study (*n* = 39). (b) Linear regression analysis between HER2 and HIF1α transcript levels in the TCGA RNA‐seq data set (*n* = 39). (c) GSEA plots of genes pre‐ranked according to Pearson’s correlation coefficient for HER2 expression levels. Significance was calculated by a two‐tailed unpaired *t* test. BC: breast cancer; GHEA: Group Herceptin in Adjuvant Therapy; GSEA: gene set enrichment analysis; HER2: human epidermal growth factor receptor 2; HIF1α: hypoxia‐inducible factor 1α; qPCR: quantitative real‐time polymerase chain reaction; TCGA: The Cancer Genomic Atlas

Enhanced glycolytic metabolism and upregulation of the expression of glycolytic enzymes in human cancer is frequently sustained and stimulated by overexpression of HIF1α (Hockel & Vaupel, 2001; Marin‐Hernandez et al., 2009; Semenza, 2003). In this context, it is reported that HIF1α expression is regulated by the HER2 signaling (Laughner et al., 2001). We, therefore, used qPCR analysis to assess a potential correlation between HER2 transcript or lactate levels and HIF1α expression (Hockel & Vaupel, 2001; Semenza, 2003). In a cohort of 39 frozen HER2‐positive BC specimens that included 18 previously tested cases, we found a positive correlation between the transcript levels of HER2 and HIF1α (*p* = 0.0092 and Pearson’s *r* = 0.4171; Figure 3a). As shown in Figure 3b, the analysis of a larger independent cohort of 160 HER2‐positive (IHC) tumor specimens selected from TCGA RNA‐seq data set provided additional evidence of a significant correlation between HER2 and HIF1α mRNA levels. In addition, GSEA was performed on the list of genes pre‐ranked according to Pearson’s correlation coefficient with HER2 transcript levels. This analysis revealed a significant enrichment of genes related to glucose metabolism and glycolysis in patients with high HER2 transcript levels (Figure 3c).

### 3.3 HER2 transcript expression correlates with lactate levels in HER2‐positive BC cell lines

**Figure 4:**
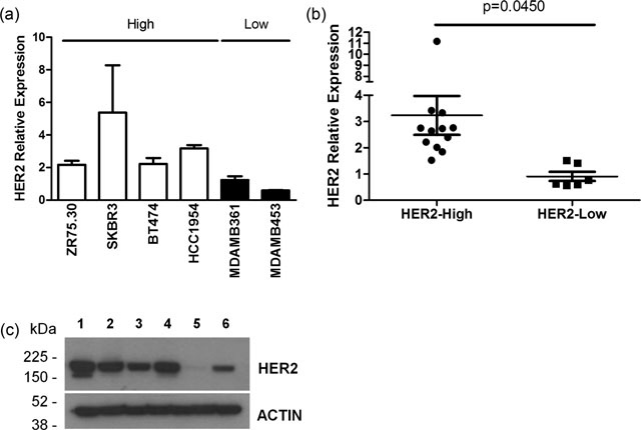
Molecular and biochemical characterization of human HER2‐positive BC cell lines. (a) Classification of HER2‐positive BC cell lines according to HER2 transcript levels (white bars: HER2‐high cells; black bars: HER2‐low cells). (b) Scatter plot of relative HER2 expression in HER2‐high versus HER2‐low cells. Each plot represents a single biological replicate (*n* = 3) of each analyzed BC cell line. Significance was calculated by a two‐tailed unpaired *t* test. (c) Western blot analysis of basal HER2 expression in human HER2‐positive BC cell lines. Protein extracts from (1) ZR75.3, (2) SKBR3, (3) BT474, (4) HCC1954, (5) MDAMB361, and (6) MDAMB453 cells were separated by 4–12% gradient SDS‐PAGE under reducing conditions and probed with an anti‐HER2 antibody. Actin was used to normalize protein loading. Autoradiographs were acquired at different exposure times to obtain optimal image resolution. BC: breast cancer; HER2: human epidermal growth factor receptor 2; SDS‐PAGE: sodium dodecyl sulfate polyacrylamide gel electrophoresis

**Figure 5:**
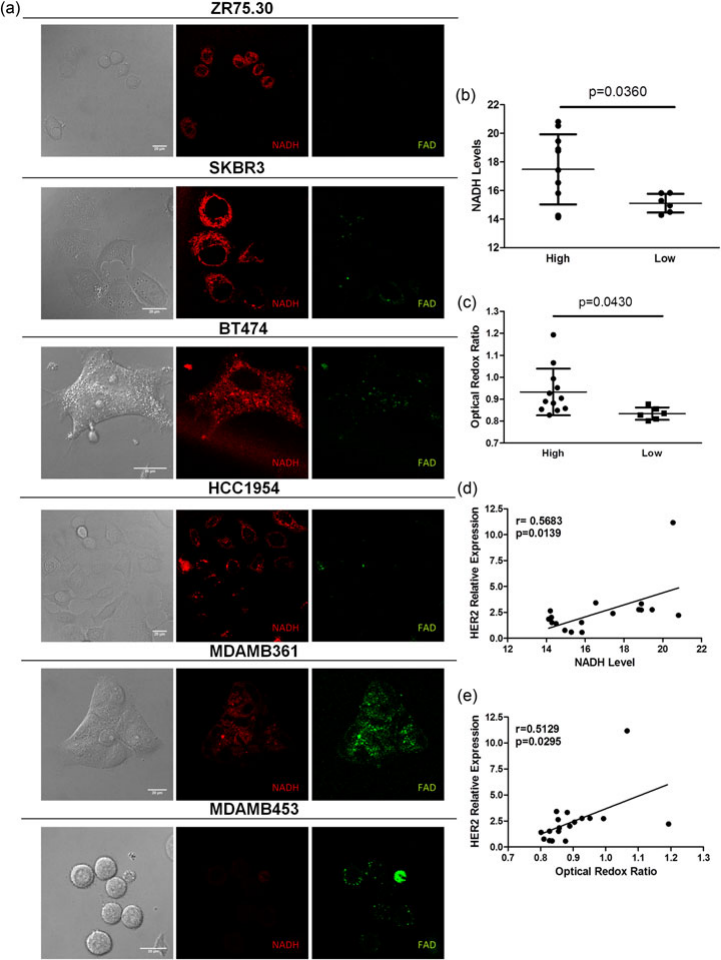
Assessment of NADH and FAD and the associations among HER2 transcript levels, NADH and the optical redox ratio in a panel of HER2‐positive BC cell lines. (a) Representative images of the fluorescence intensity of NADH and FAD evaluated by confocal microscopy in ZR75.30, SKBR3, BT474, HCC1954, MDAMB361, and MDAMB453 cells. (b) Scatter plot of NADH levels in HER2‐high versus HER2‐low BC cell lines. The data are presented as the mean ± *SD*. Each plot represents a single biological replicate (*n* = 3) of each analyzed BC cell line. (c) Scatter plot of the optical redox ratio in HER2‐high versus HER2‐low BC cell lines. The data are presented as the mean ± *SD*. Each plot represents a single biological replicate (*n* = 3) of each analyzed BC cell line. (d) Linear regression analysis between HER2 transcript levels and NADH. Each plot represents a single biological replicate (*n* = 3) of each analyzed BC cell line. (e) Linear regression analysis between HER2 transcript expression levels and the optical redox ratio. Each plot represents a single biological replicate (*n* = 3) of each analyzed BC cell line. Significance was calculated by a two‐tailed unpaired *t* test. BC: breast cancer; FAD: flavin adenine dinucleotide; NADH: nicotinamide adenine dinucleotide

**Figure 6:**
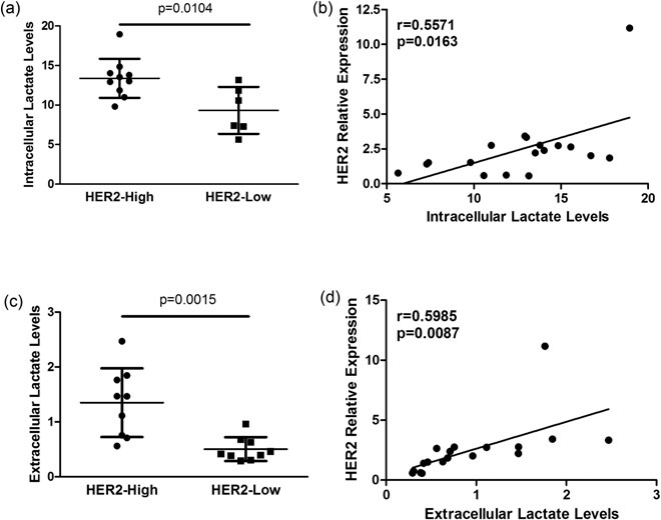
Association between HER2 transcript expression and intracellular and extracellular lactate levels. (a) Scatter plot of intracellular lactate levels in HER2‐high versus HER2‐low BC cell lines. Each plot represents a single biological replicate (*n* = 3) of each analyzed BC cell line. (b) Linear regression analysis between HER2 transcript levels and intracellular lactate levels. (c) Scatter plot of extracellular lactate levels in HER2‐high versus HER2‐low BC cell lines. Each plot represents a single biological replicate (*n* = 3) of each analyzed BC cell line. (d) Linear regression analysis between HER2 transcript levels and extracellular lactate levels. Each plot represents a single biological replicate (*n* = 3) of each analyzed BC cell line. Significance was calculated by a two‐tailed unpaired *t* test. BC: breast cancer; HER2: human epidermal growth factor receptor 2

To confirm the association between HER2 addiction and glycolytic metabolism and to exclude a possible role of cells in the tumor microenvironment in regulating intratumor lactate content, we performed in vitro analyses using the following HER2‐positive tumor cell lines: ZR75.30, SKBR3, BT474, HCC1954, MDAMB361, and MDAMB453. Based on HER2 mRNA expression (Figure 4a), we classified these cell lines as HER2‐high (ZR75.30, SKBR3, BT474, and HCC1954) or HER2‐low (MDAMB361 and MDAMB453; Figure 4b). Western blot analysis showed that HER2 protein levels were considerably higher in cells expressing higher HER2 mRNA levels (i.e., ZR75.30, SKBR3, BT474, and HCC1954 cells; Figure 4c, lanes 1–4) than in cells with lower HER2 mRNA expression (i.e., MDAMB361 and MDAMB453; Figure 4c, lanes 5 and 6). To characterize the balance between glycolytic metabolism and oxidative phosphorylation (i.e., tricarboxylic acid [TCA] cycle activation) in these cell models, we assessed the cellular energetic status by optical imaging. Specifically, we measured the intratumor autofluorescence of NADH, a reduced coenzyme that is produced during glycolysis and oxidized during mitochondrial cell respiration, and FAD, an oxidized coenzyme that is highly expressed in cells with an active TCA cycle and oxidative phosphorylation (Figure 5a). The intracellular balance between glycolysis and oxidative metabolism was calculated using the optical redox ratio between NADH and FAD fluorescence intensity: more glycolysis is active more NADH:FAD ratio is high. Conversely, TCA cycle activation is associated with a lower NADH:FAD ratio. Confocal microscopy analyses showed a significant enrichment of intracellular NADH (Figure 5b), a higher optical redox ratio (Figure 5c) in HER2‐positive BC cell lines characterized by higher HER2 transcript levels. Furthermore, we observed a significant direct correlation between HER2 mRNA and NADH levels (Figure 5d), between HER2 mRNA levels and the optical redox ratio (Figure 5e). These findings indicate that HER2 overexpression is associated with the upregulation of glycolytic metabolism. Then, we analyzed the global metabolic content of HER2‐positive BC cell lines by high‐resolution MRS; in accordance with previous results, we detected a significant enrichment of intracellular lactate levels in BC cell lines with high HER2 mRNA expression (Figure 6a). We also found a direct correlation between HER2 transcript levels and lactate content (Figure 6b). Since glycolytic cancer cells produce and release lactate in the extracellular microenvironment, we measured lactate levels in conditioned media from matched HER2‐positive BC cell lines (Supporting Information Figure 3). This analysis revealed significantly higher lactate levels in conditioned media from BC cell lines with higher HER2 mRNA expression. In contrast, intracellular levels of succinate (Supporting Information Figure 4), a metabolite produced in the TCA cycle and associated with active oxidative phosphorylation, were significantly higher in HER2‐positive BC cell lines characterized by lower HER2 mRNA levels, and HER2 mRNA expression was inversely associated with intracellular succinate content. Taken together, our data support the hypothesis that high HER2 levels are associated with the upregulation of glycolysis compared with oxidative mitochondrial phosphorylation, resulting in lactate production and excretion by cancer cells.

## 4 DISCUSSION

In this study, we provide the first evidence that intratumor lactate levels and glycolytic metabolism reflect addiction of HER2‐positive BC models to HER2 signaling, and are associated with higher HER2 mRNA levels. We confirmed these findings through gene expression and metabolic analyses of HER2‐positive cancer cell lines, transgenic models, and human tumor specimens.

Although the introduction of anti‐HER2 therapies has dramatically improved the cure rate and survival expectancy of patients with early stage and advanced‐stage HER2‐positive BC, respectively, tumor sensitivity to anti‐HER2 treatments is highly heterogeneous (Loibl & Gianni, 2017). In recent years, specific gene expression signatures have been found to be associated with the tumor response to trastuzumab (Castagnoli et al., 2014; Prat et al., 2014; Triulzi et al., 2015). HER2 mRNA levels have also been proposed to reflect HER2 expression and to predict trastuzumab efficacy better than the currently used IHC and FISH techniques (Pogue‐Geile et al., 2013; Prat et al., 2014; Triulzi et al., 2015). Our in silico data strongly support this hypothesis and confirm that HER2 mRNA levels represent an easy‐to‐measure, low‐cost and semiquantitative biomarker that reflects the HER2 addiction status and trastuzumab susceptibility. However, HER2 mRNA quantification will be difficult to implement in standard clinical practice due to concerns with the quality of tumor specimen fixation, embedding and deparaffinization, which are crucial steps for the reliable quantitative analysis of HER2 mRNA levels.

Previous studies have reported enhanced glycolytic metabolism in HER2‐positive BC and HER2‐mediated expression of glycolysis‐related genes (Walsh et al., 2013). In particular, HER2 signaling is known to upregulate LDH‐A and HIF1α expression (Zhao et al., 2009), while HER2 downregulation or pharmacologic inhibition with trastuzumab reverses the glucose dependency and inhibits glycolytic metabolism in HER2‐positive BC cells (Walsh et al., 2013).

However, to the best of our knowledge, the upregulation of glycolysis has not been correlated with tumor response to anti‐HER2 agents. The major finding of our study is the association between enhanced glycolytic metabolism and addiction to HER2 signaling in HER2‐positive BC. We also found an association between the activation of glycolysis and higher HER2 mRNA levels in tumor specimens from patients who benefited from adjuvant trastuzumab therapy. To exclude the possibility that stromal and infiltrating immune cells were responsible for the enhanced lactate production in the analyzed tissue specimens (Romero‐Garcia et al., 2016), we confirmed correlation between HER2 mRNA expression and lactate levels in a panel of HER2‐positive BC cell lines. Taken together, our results suggest a strong correlation between HER2 mRNA and protein levels and activation of the glycolysis pathway in HER2‐positive BC, which could be mediated by overexpression of HIF1α. Since glycolytic pathway activation is positively correlated with HER2 mRNA and protein levels, markers of glycolysis such as intratumor lactate levels could be used as a quantitative, noninvasive and reliable biomarker of sensitivity to anti‐HER2 treatments.

The potential role of lactate quantification in predicting tumor sensitivity to anticancer treatment and patient survival has been previously investigated. For example, Blatt et al. (2016) reported that lactate levels in head and neck squamous cell carcinoma specimens inversely correlated with patient OS and PFS after surgery and radiation therapy. The measurement of intratumor lactate levels with noninvasive methodologies, such as nuclear magnetic resonance, was previously proposed as a method to identify key biomolecules and molecular changes associated with cancer aggressiveness and treatment and to improve clinical cancer care (Haris et al., 2015). Our study adds to this knowledge by establishing a link between intratumor lactate levels and addiction to HER2 signaling in HER2‐positive BC that predicts the benefit from anti‐HER2 treatments.

The strengths of our study include the following: the identification of a metabolic signature associated with addiction to HER2 signaling, thus paving the way for noninvasive intratumor lactate measurements to identify patients who will benefit from anti‐HER2 therapies; and (b) the consistency of our results in preclinical models (both in vitro and in vivo) and in tumor specimens. The major limitation of the study is the small number of tumor specimens analyzed in the validation patient cohort.

The identification of lactate quantification as a novel, noninvasive, and reliable metabolic biomarker of addiction to HER2 signaling may improve the selection of HER2‐positive BC patients who are more likely to benefit from standard anti‐HER2 treatments, while unselected patients are steered towards enrollment in clinical trials. Larger studies are required to confirm that intratumor lactate is a *bona fide* indicator of HER2 mRNA expression levels and of the clinical benefit of anti‐HER2 treatments in the adjuvant and metastatic settings for patients with HER2‐positive BC.

## CONFLICTS OF INTERESTS

All authors declare that there are no conflicts of interest.

## AUTHORS’ CONTRIBUTIONS

L. C. contributed to the study concept and design; acquisition of data and materials; analysis and interpretation data; drafting of the manuscript; and statistical analysis. E. I. contributed to the acquisition of data; analysis and interpretation of metabolic data; and statistical analysis. M. D. contributed to the acquisition of data; analysis and interpretation of microarray data; and statistical analysis. A. K. and S. F. contributed to the acquisition of data and materials. R. C. contributed to the acquisition and interpretation of metabolic data. P. N. contributed to the acquisition of materials; and the critical revision. C. V. contributed to the drafting and editing of the manuscript; and the critical revision. M. D. N. contributed to the critical revision. D. M. contributed to the acquisition of data; analysis and interpretation data; and the critical revision. E. T. contributed to the study concept and design; and the critical revision. S. M. P. contributed to the study concept and design; study supervision; obtained funding; drafting of the manuscript; and the critical revision. All authors read and approved the final manuscript.

## Supporting information


**Supplementary Figure 1**. ( **A and B)** Lapatinib‐mediated antitumor activity in parental FVB mice following the orthotopic injection of MI6 or WTHER2_1 tumor cells. Tumor‐bearing mice were treated *per os* with Lapatinib (▴, 200 mg/kg daily until sacrifice) or diluent DMSO (●, 150 μl daily until sacrifice) in the presence of evident disease. Data are presented as the mean±SEM. ***p<0.001, unpaired t‐test

**Supplementary Figure 2**. Box plots showing HER2 mRNA expression levels [ERBB2 log2(intensity)] measured by microarray analysis in 53 HER2‐positive BC patients treated with adjuvant trastuzumab (GHEA cohort, GSE55348) distributed according to PAM50 BC classification

**Supplementary Figure 3**. Extracellular lactate levels in conditioned medium from ZR75.30, SKBR3, BT474, HCC1954, MDAMB453 and MDAMB361 cells were evaluated by a blood gas analyzer

**Supplementary Figure 4. A)** Scatter plot of intracellular succinate levels in HER2‐high vs HER2‐low BC cell lines. Each plot represents a single biological replicate (n=3) of each analyzed BC cell line. **B)** Linear regression analysis between HER2 transcript levels and intracellular succinate levels. Each plot represents a single biological replicate (n=3) of each analyzed BC cell line. Significance was calculated by a two‐tailed unpaired *t*‐test

**Supplementary Table 1**. HER2‐positive BC patient pathobiological and clinical characteristics
