# Supplementary figures and images for: Intratumor lactate levels reflect HER2 addiction status in HER2‐positive breast cancer
Source: J Cell Physiol. 2018 Aug 21;234(2):1768–79. doi: 10.1002/jcp.27049 (PMC6282573; doi:10.1002/jcp.27049)

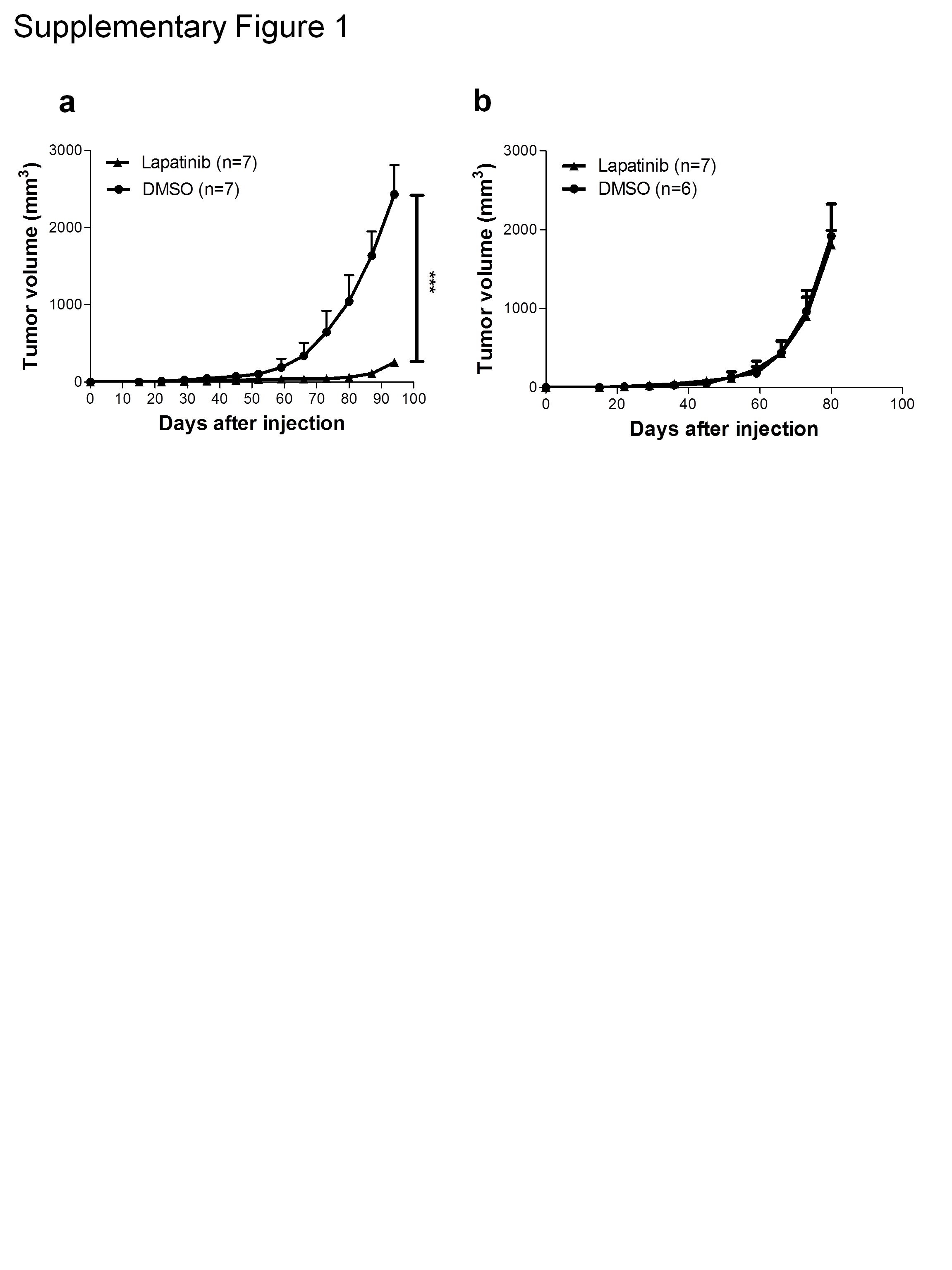

Supplement: Supplementary file 1 — Supplementary Figure 1. ( A and B) Lapatinib‐mediated antitumor activity in parental FVB mice following the orthotopic injection of MI6 or WTHER2_1 tumor cells. Tumor‐bearing mice were treated per os with Lapatinib (▴, 200 mg/kg daily until sacrifice) or diluent DMSO (●, 150 μl daily until sacrifice) in the presence of evident disease. Data are presented as the mean±SEM. ***p<0.001, unpaired t‐test [file JCP-234-1768-s001.tif]

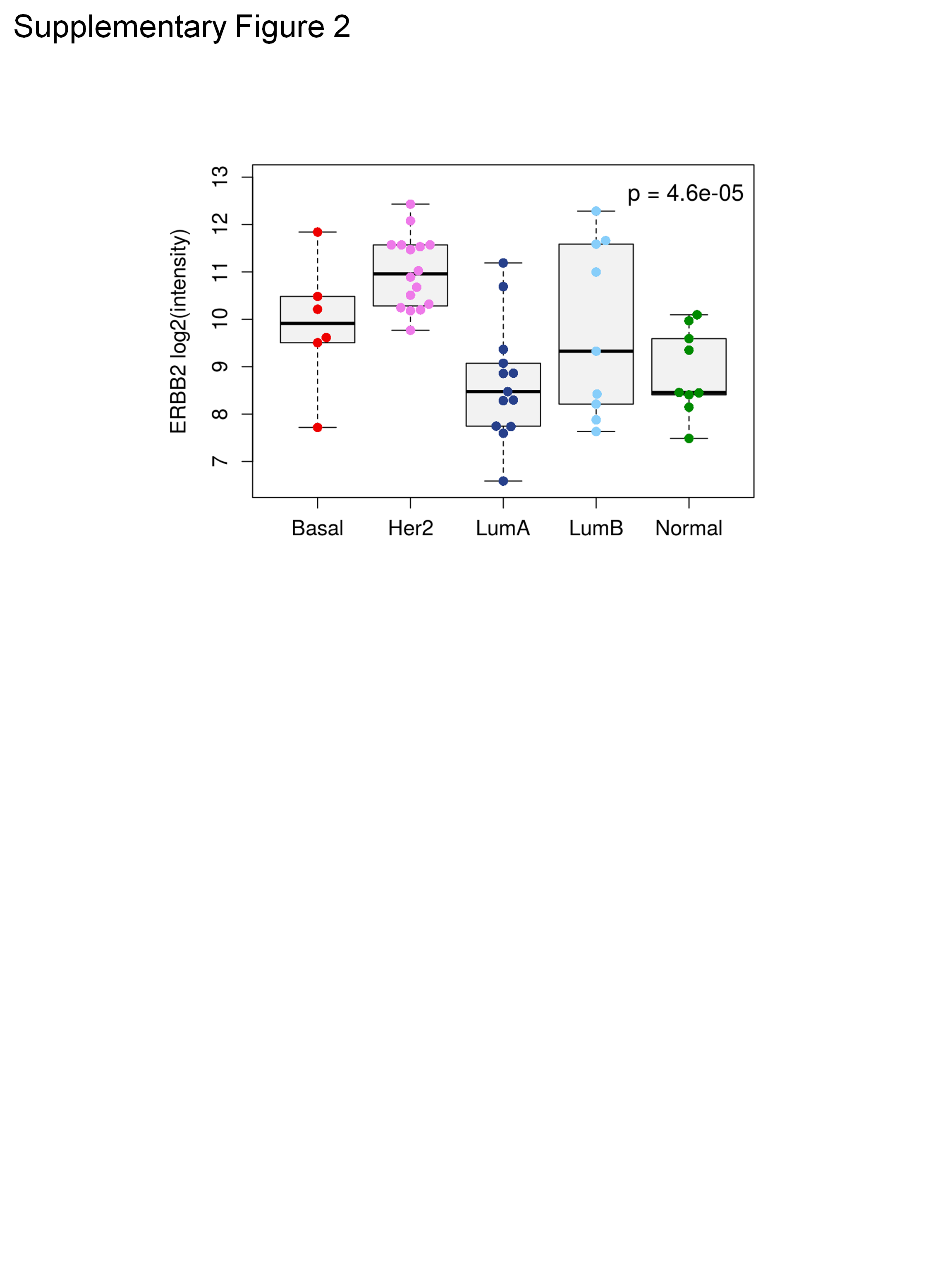

Supplement: Supplementary file 2 — Supplementary Figure 2. Box plots showing HER2 mRNA expression levels [ERBB2 log2(intensity)] measured by microarray analysis in 53 HER2‐positive BC patients treated with adjuvant trastuzumab (GHEA cohort, GSE55348) distributed according to PAM50 BC classification [file JCP-234-1768-s002.tif]

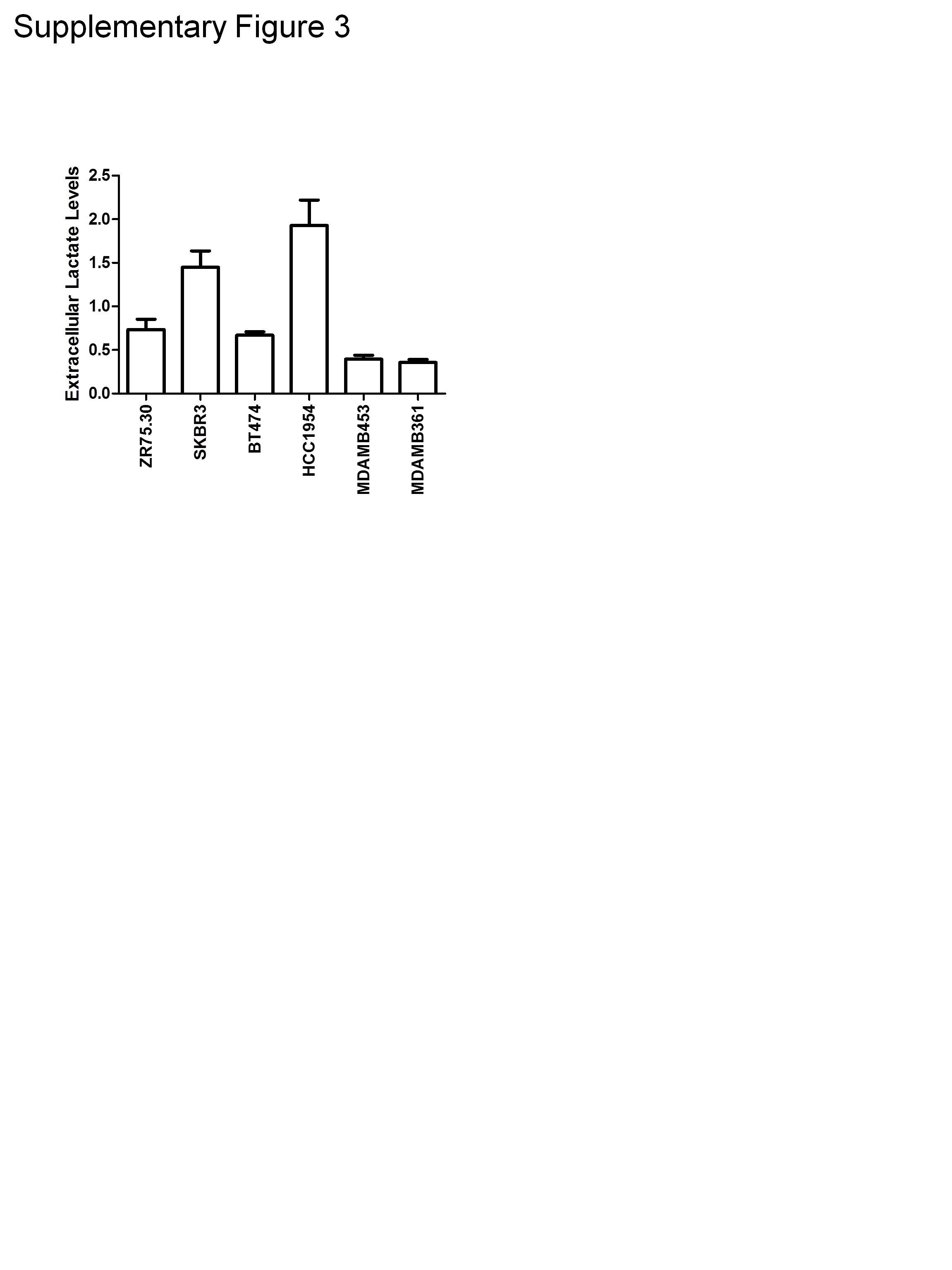

Supplement: Supplementary file 3 — Supplementary Figure 3. Extracellular lactate levels in conditioned medium from ZR75.30, SKBR3, BT474, HCC1954, MDAMB453 and MDAMB361 cells were evaluated by a blood gas analyzer [file JCP-234-1768-s003.tif]

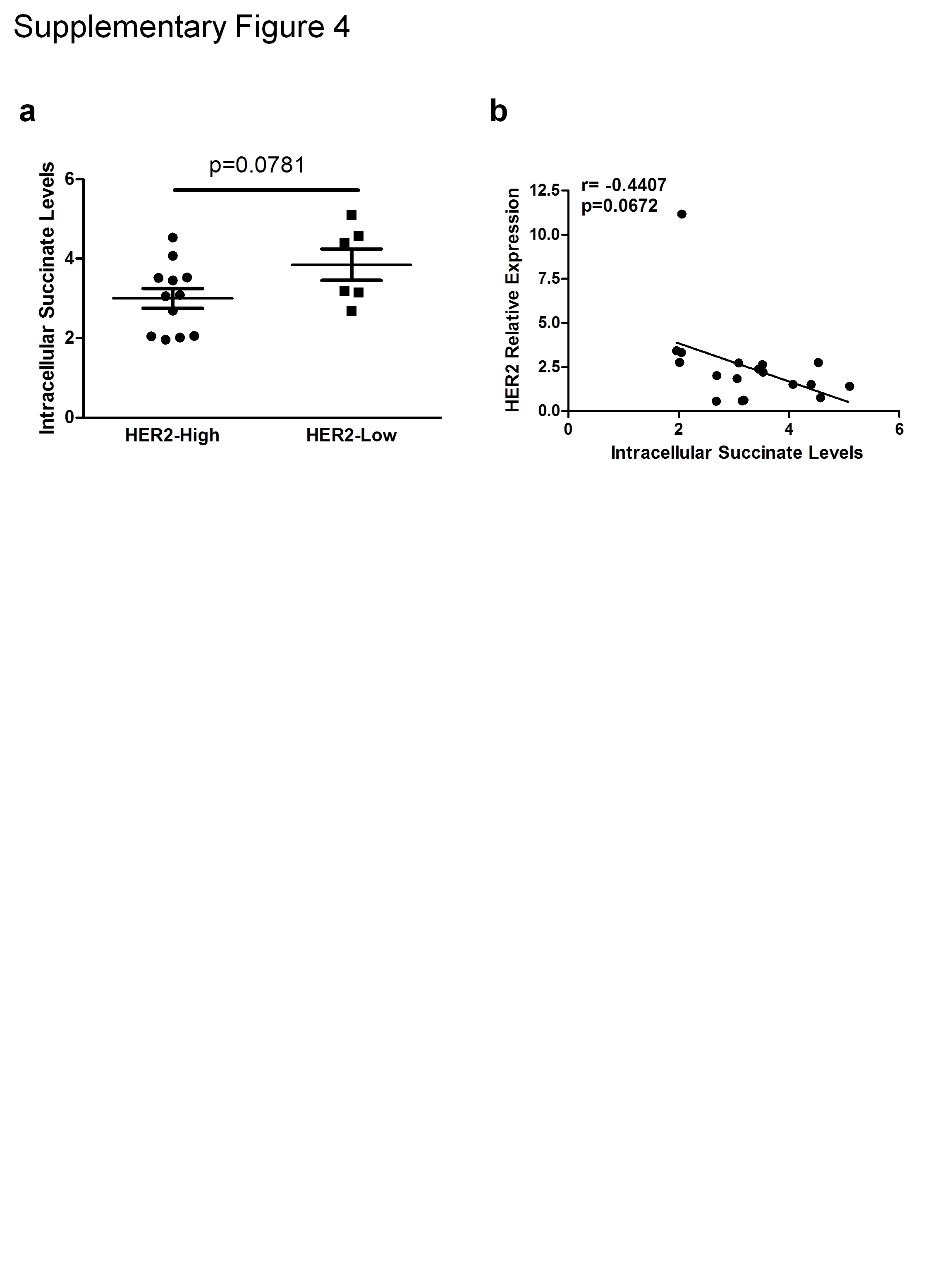

Supplement: Supplementary file 4 — Supplementary Figure 4. A) Scatter plot of intracellular succinate levels in HER2‐high vs HER2‐low BC cell lines. Each plot represents a single biological replicate (n=3) of each analyzed BC cell line. B) Linear regression analysis between HER2 transcript levels and intracellular succinate levels. Each plot represents a single biological replicate (n=3) of each analyzed BC cell line. Significance was calculated by a two‐tailed unpaired t‐test [file JCP-234-1768-s004.tif]
