# Supplementary material for: Intratumor lactate levels reflect HER2 addiction status in HER2‐positive breast cancer
Source: J Cell Physiol. 2018 Aug 21;234(2):1768–79. doi: 10.1002/jcp.27049 (PMC6282573; doi:10.1002/jcp.27049)
Supplement: Supplementary file 5 — Supplementary Table 1. HER2‐positive BC patient pathobiological and clinical characteristics [file JCP-234-1768-s005.docx]

| Characteristics | No./tot (%) |
| --- | --- |
| Age at diagnosis:  <50  ≥50  Missing | 14/39 (36)  21/39 (54)  4/39 (10) |
| Grade:  I-II  III  Missing | 14/39 (36)  20/39 (51)  5/39 (13) |
| Nodal status:  Positive  Negative  Missing | 28/39 (72)  7/39 (18)  4/39 (10) |
| Tumor size:  T1  T2,T3,T4  Missing | 16/39 (41)  18/39 (46)  5/39 (13) |
| ER:  Negative  Positive  Missing | 17/39 (44)  18/39 (46)  4/39 (10) |
| PgR  Negative  Positive  Missing | 16/39 (41)  19/39 (49)  4/39 (10) |
| HER2 IHC  2+  3+  Missing | 2/39 (5)  33/39 (85)  4/39 (10) |
| Neoadjuvant therapy  Yes  No  Missing | 1/39 (2)  30/39 (77)  8/39 (21) |

**Supplementary Table 1**
